# Prognostic impact of interstitial lung abnormalities in lung cancer: a systematic review and meta-analysis

**DOI:** 10.3389/fonc.2024.1397246

**Published:** 2024-05-10

**Authors:** Xian-Liang Tang, Yin-Bo Sun, Xiao-Tong Guo, Sheng-Zhao Yang, Wen-Ping Zhang

**Affiliations:** ^1^ Department of Thoracic Surgery, Heping Hospital Affiliated to Changzhi Medical College, Changzhi, Shanxi, China; ^2^ Department of Rehabilitation, Heping Hospital Affiliated to Changzhi Medical College, Changzhi, Shanxi, China

**Keywords:** lung cancer, meta-analysis, interstitial lung abnormality, prognosis, evidence-based medicine

## Abstract

**Background:**

Newly identified as a radiological concept, interstitial lung abnormalities (ILA) is emerging as a prognostic factor for lung cancer. Yet, debates persist regarding the prognostic significance of ILA in lung cancer. Our inaugural meta-analysis aimed to investigate the correlation between ILA and lung cancer outcomes, offering additional insights for clinicians in predicting patient prognosis.

**Methods:**

Articles meeting the criteria were found through PubMed, the Cochrane Library, EMBASE, and Web of Science by February 29, 2024. The outcomes evaluated were the survival rates such as overall survival (OS), disease-free survival (DFS), progression-free survival (PFS), and cancer-specific survival (CSS).

**Results:**

A total of 12 articles with 4416 patients were included in this meta-analysis. The pooled results showed that lung cancer patients with interstitial lung abnormalities had an inferior OS (n=11; HR=2.22; 95% CI=1.68-2.95; P<0.001; I^2^ = 72.0%; Ph<0.001), PFS (n=3; HR=1.59; 95% CI=1.08-2.32; P=0.017; I^2^ = 0%; Ph=0.772), and CSS (n=2; HR=4.00; 95% CI=1.94-8.25; P<0.001; I^2^ = 0%; Ph=0.594) than those without, however, the ILA was not significantly associated with the DFS (n=2; HR=2.07; 95% CI=0.94-7.02; P=0.066; I^2^ = 90.4%; Ph=0.001). Moreover, lung cancer patients with ILA were significantly correlated with male (OR=2.43; 95% CI=1.48-3.98; P<0.001), smoking history (OR=2.11; 95% CI=1.37-3.25; P<0.001), advanced age (OR=2.50; 95% CI=1.56-4.03; P<0.001), squamous carcinoma (OR=0.42; 95% CI=0.24-0.71; P=0.01), and EGFR mutation (OR=0.50; 95% CI=0.32-0.78; P=0.002). The correlation between ILA and race, stage, ALK, however, was not significant.

**Conclusion:**

ILA was a availability factors of prognosis in patients with lung cancers. These findings highlight the importance of early pulmonary fibrosis, namely ILA for prognosis in patients with lung cancer, and provide a partial rationale for future clinical work.

## 1 Introduction

Lung cancer still stands as one of the malignancies with the highest mortality and morbidity globally (1, 2). Despite advancements in treatment options for lung cancer, survival rates have increased from previous levels, yet the overall prognosis remains grim. As a result, there is a continuous effort to investigate the various factors influencing the prognosis of individuals diagnosed with lung cancer.

ILA were initially detected as a radiologic discovery based on incidental CT findings of abnormalities impacting over 5% of lung regions, including ground-glass or reticular abnormalities, lung distortion, traction bronchiectasis, honeycombing, and non-emphysematous cysts (3). ILA is considered to be an early stage of fibrotic lung disease (4), and there is a strong correlation between ILA and lung cancer (5). Numerous studies (5, 6) indicating an increased occurrence of lung cancer and heightened pulmonary complications following cancer therapy in ILA patients. Additionally, some studies (7–14) suggest that combined ILA significantly worsens the prognosis in lung cancer patients. Conversely, conflicting studies (15–18) have shown no clear correlation between ILA and survival outcome among lung cancer patients. These results are inconclusive, leading to a continued debate over the association of ILA with lung cancer prognosis. Thus, we conducted a comprehensive meta-analysis of existing literature regarding the prognosis of lung cancer patients with ILA.

## 2 Methods

### 2.1 Literature search strategies

The current meta-analysis accompanied the PRISMA statement (19). On February 29, 2024, PubMed, EMBASE, Web of science, and the Cochrane Library were retrieved for English language studies. The search term consists of a combination of the following medical subject heading (MeSH) and free text words: (lung carcinoma OR lung adenocarcinoma OR lung neoplasm OR lung cancer) AND (interstitial lung abnormality OR ILA OR subclinical ILD OR early ILD) AND (prognosis OR prognoses OR survival OR outcome) AND (computed tomography). Additionally, we manually retrieved the reference lists of the publications that qualified. Details of the literature search are presented in **Supplementary Table 1**.

### 2.2 Inclusion and exclusion criteria

After the initial search and removal of the duplicates, screening of the title and abstract was done by 2 independent investigators (TXL and GXT). Full texts were retrieved for studies that satisfied the inclusion criteria. Any disagreements were resolved through discussion. Bibliography sections of the included studies were further searched for additional relevant papers.

Inclusion Criteria

Utilizing the Population-Intervention-Control-Outcome-Study (PICOS) framework, the inclusion criteria for the current study were developed as follows: patient population consisting of individuals diagnosed with lung carcinoma through pathological or histological means; Exposure-intervention involving a computed tomography (CT) scan of the lungs identifying ILA, with a positive diagnosis by a radiologist; Control group comprised of individuals whose lung CT scans showed no signs of ILA or interstitial lung disease; Desired outcomes consisted of English-language studies examining the relationship between ILA and prognosis in lung cancer patients, including OS, DFS, PFS, and CSS, as well as hazard ratio (HR) and 95% confidence interval (CI) related to patient survival; And study design encompassing both retrospective and prospective English-language publications. Classification of patients into ILA group and non-ILA group for the intervention and control groups was based on the definition of ILA identified in chest CT scans.

Exclusion Criteria

Conference abstracts, case reports, or comments were excluded. Studies that compared only the clinicopathologic features and not the prognostic value of ILA were excluded.

### 2.3 Data extraction and quality assessment

The main focus of data extraction was on the author, year of publication, region of study, design of the study, period of study, size of the sample, ILA incidence, types of cancer, treatment methods, duration of follow-up, and study outcomes. The quality of observational studies was assessed using the Newcastle-Ottawa Scale (NOS) score (20). Literature was considered high-quality if it scored above six. Two authors (GXT and TXL) independently double-checked all of the aforementioned steps, and any discrepancies were resolved by a third author (YSZ).

### 2.4 Statistical methods

Stata 16.0 was utilized for the statistical analysis. OS is determined as the period from the initiation of therapy until decease, irrespective of the reason. A fatality linked to lung cancer or a respiratory ailment is counted as a cause-specific decease, and the period from the initiation of therapy until a cause-specific decease is designated as CSS. Within this meta-analysis, DFS and RFS in the included studies were jointly denoted as DFS, which is characterized as the duration from surgery until the reappearance of the disease or decease from any cause (event). The combined HR and 95%CI were utilized to assess the connection between ILA and prognosis in lung cancer patients. Multivariate HR and 95% CI data were directly obtained from the study. In cases where multivariate HR was not provided, univariate HR data was extracted. An HR greater than 1 (with a 95%CI not overlapping 1) suggested that ILA was linked to a poorer prognosis in patients, rather than the opposite being true. In terms of examining the relationship between ILA and clinical and pathological characteristics of lung cancer patients, the combined odds ratio (OR) and 95%CI were employed. An OR greater than or less than 1 (with a 95%CI not overlapping 1) indicated a correlation between ILA and clinicopathological features in lung cancer patients, and not the contrary. The chisquared test was employed to calculate the statistical heterogeneity. A random effect model was applied when P<0.1 and I^2^>50% indicated high heterogeneity; otherwise, the fixed effect model was utilized. Egger’s tests were conducted to assess publication bias, and in cases of significant bias, the trim-and-fill method was applied to adjust the results. Sensitivity analysis was carried out to evaluate result stability, with each study being excluded independently.

## 3 Results

### 3.1 Characteristics of studies

After the initial search, 57 duplicate studies were removed. Then there were 285 articles deleted after carefully reading the titles and abstracts. Later, the full texts of the remaining 45 articles were further assessed.

**Figure 1:**
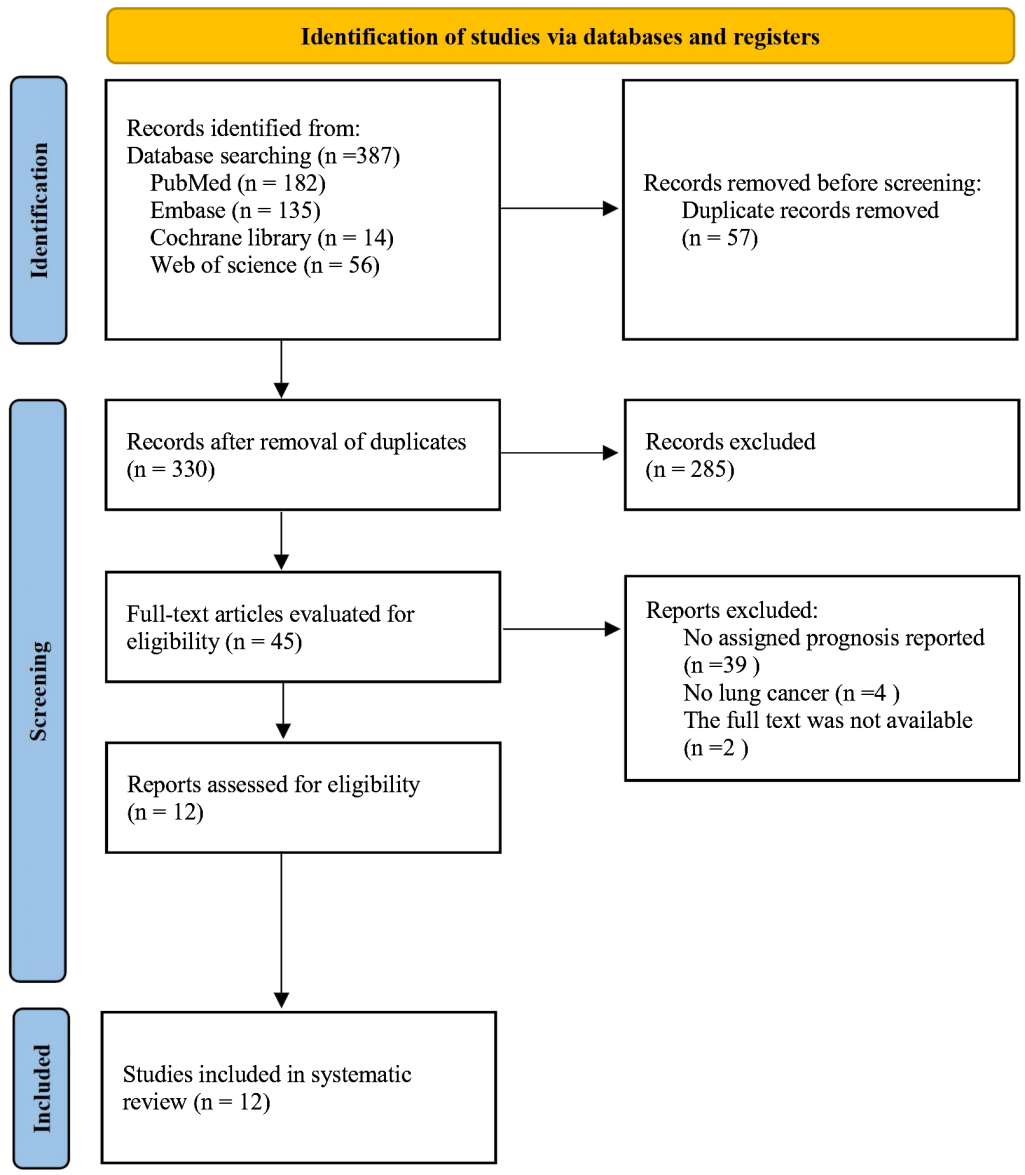
Flow diagram for selection of studies.

12 articles (7–18) involving 4416 patients were ultimately included. The PRISMA flow diagram is provided in **Figure 1**. The main characteristics of the studies included are shown in **Table 1**. All the 12 studies were retrospective. Four studies were from Korea and Japan, respectively, three studies were carried out in USA, the remaining one in China. Ten studies involved the treatment of NSCLC, one studies included small-cell lung cancer (SCLC), and one study had both NSCLC and SCLC arms. The sample sizes varied from 73 to 1,524. The incidence rate of ILA ranged from 3.93% to 37.87%, with an average incidence of 14.77%. The NOS scores for the 12 articles ranged from 6 to 8, which represented a low risk of bias (**Table 1**).

**Table 1:** Baseline characteristics and quality assessment of the included studies.

Study	year	region	sample	stage	Follow-up time (months)	Outcome	treatment	Analysis	NOS
Ito et al. (16)	2023	Japan	175	IIA-IIIC	2.5 (0.6–12.1)	OS/PFS	Chemoradiotherapy	U/U	6
Zhu et al. (14)	2023	China	765	I-IV	N/A	OS	Combination therapy	M	8
Nishino et al. (19)	2015	USA	120	IV	18.4 (2.1-23.7)	OS	Chemotherapy	M	7
Kashihara et al. (17)	2023	Japan	113	IIB-IIIC	24 (5–47)	OS/PFS/CSS	Chemoradiotherapy	M/M/M	8
Araki et al. (8)	2019	USA	484	IV	N/A	OS	Chemotherapy	M	7
Hida et al. (10)	2021	USA	231	I	32.2 (0.4-189)	OS	Combination therapy	M	8
Jeong et al. (12)	2023	Korea	228	IA	N/A	CSS	Surgery	M	8
Ahn et al. (7)	2023	Korea	1524	I-III	38.5 (0.7–84.4)	OS/RFS	Surgery	M/M	7
Higo et al. (15)	2019	Japan	77	IIIA-IIIB	N/A	OS	Chemoradiotherapy	U	7
Gu et al. (9)	2019	Korea	382	I-III	56.2	OS/DFS	Surgery	M/M	8
Kobayashi et al. (18)	2021	Japan	149	I-III	60.4(12.4-120.6)	OS/PFS	Chemoradiotherapy	M/U	8
Im et al. (11)	2023	Korea	250	I-III	52.9 (28.3-60.0)	OS	Surgery	M	7

OS, Overall survival; PFS, progression-free survival; DFS, disease-free survival; CSS, cause-specific mortality; M, multivariate analysis; U, univariate analysis; NOS, Newcastle-Ottawa Scale score.

### 3.2 ILA and overall survival

**Figure 2:**
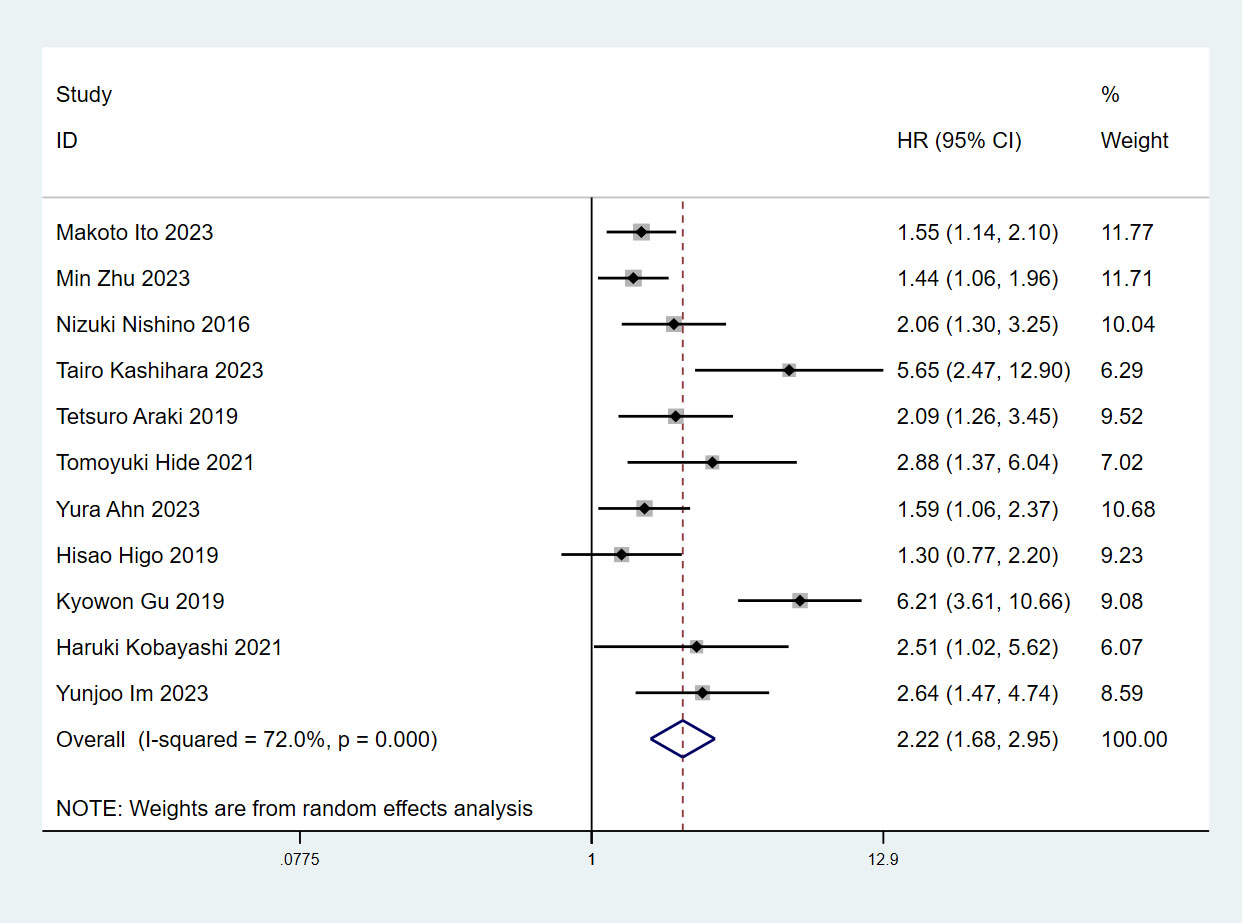
Forest plots of the association between ILA and the overall survival of patients with lung cancer.

In total, 11 articles (7–11, 13–18) involving 4188 patients explored the association between ILA and OS in lung cancer patients. The pooled HR was (n=11; HR=2.22; 95% CI=1.68-2.95; P<0.001; I^2^ = 72.0%; Ph<0.001), implying that the lung cancer patients with ILA raised death risk by 122% compared to those without (**Figure 2**).

Since there was significant heterogeneity, a random effects model was used (I^2^ = 73.9%, P<0.001). We then conducted subgroup analyses based on study region, sample size, cancer types, treatment and analysis. The results were consistent with the above findings (**Table 2**, **Supplementary Figures S1–5**).

**Table 2:** Subgroup analysis of the association between ILA and overall survival in patients with lung cancer.

Subgroup	Number of study	Heterogenicity		HR(95%CI)	P value	Effect model
		I²(%)	Ph			
**OS**	11	72.00%	<0.001	2.22(1.68,2.95)	<0.001	Random
Treatment						
Chemoradiotherapy	4	70.8	0.016	2.09(1.22,3.58)	0.007	Random
Chemotherapy	2	0	0.962	2.07(1.48,2.90)	<0.001	Random
Surgery	3	87.3	<0.001	2.93(1.29,6.63)	0.01	Random
Region						
Japan	4	70.8	0.016	2.09(1.22,3.58)	0.007	Random
USA	3	0	0.73	2.19(1.61,2.98)	<0.001	Random
Korea	3	87.3	<0.001	2.93(1.29,6.63)	0.01	Random
China	1	–	–	1.44(1.06,1.96)	0.021	–
Tumor histology						
NSCLC	9	75.8	<0.001	2.35(1.71,3.23)	<0.001	Random
SCLC	1	–	–	2.51(1.07,5.89)	0.035	–
Sample size						
<200	5	62.6	0.03	2.03(1.37,3.00)	<0.001	Random
≥200	6	79.3	<0.001	2.39(1.68,2.95)	0.001	Random
Analysis						
Univariable	2	0	0.576	1.48(1.14,1.93)	0.004	Random
Multivariable	9	73.2	<0.001	2.52(1.79,3.54)	<0.001	Random
**PFS**	3	0	<0.001	1.59(1.08,2.32)	0.017	Fixed
**DFS**	2	90.4	0.001	2.07(0.94,7.02)	0.066	Random
**CSS**	2	0	0.594	4.00(1.94,8.25)	<0.001	Fixed

OS, overall survival; PFS, progression-free survival; DFS, disease-free survival; CSS, cause-specific mortality; HR, hazard ratio; CI, confidence interval; Ph , P values of Q test for heterogeneity test.

### 3.3 ILA and progression-free survival

**Figure 3:**
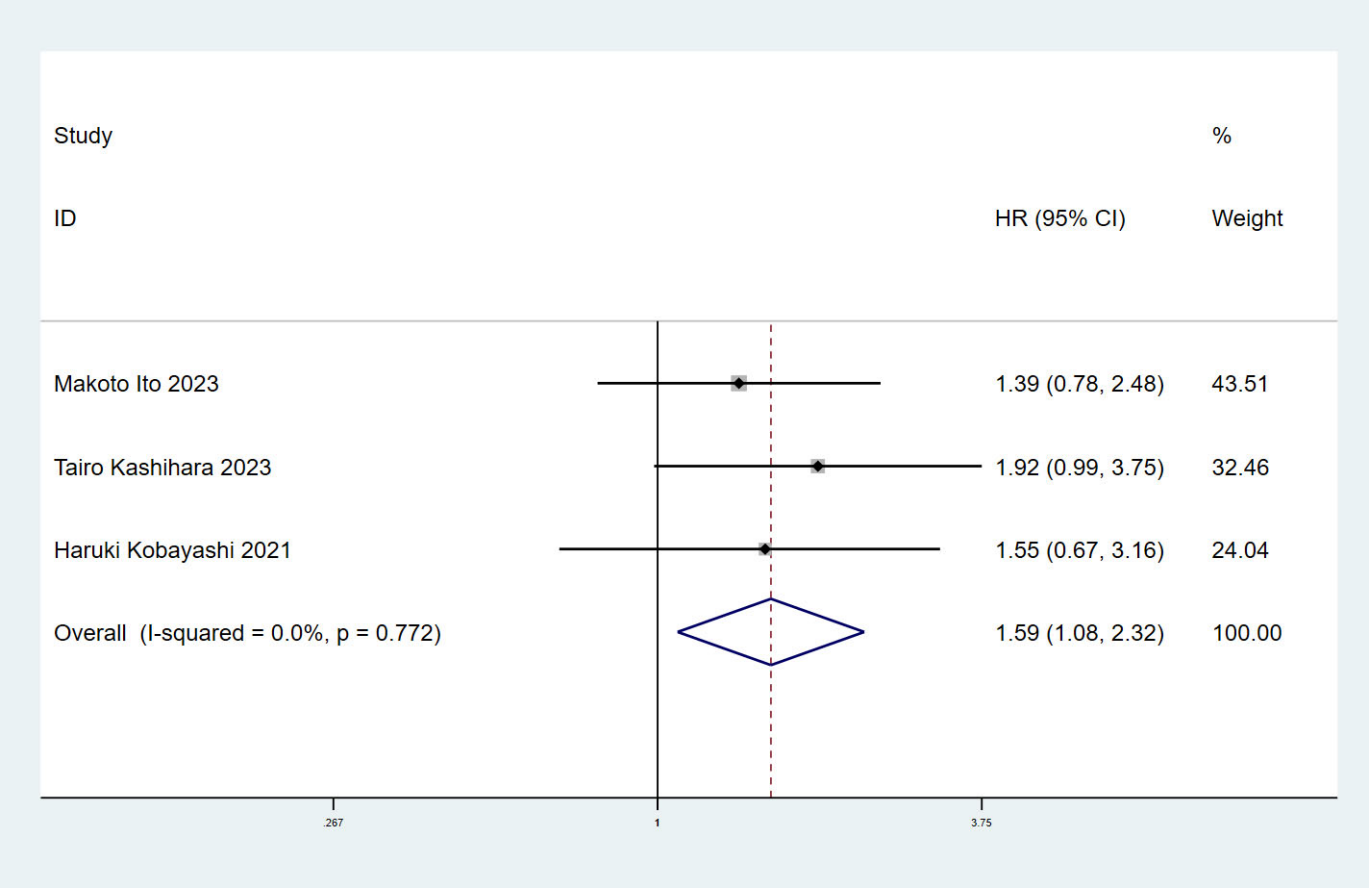
Forest plots of the association between ILA and the progression-free survival of patients with lung cancer.

The relationship between ILA and PFS was examined using prognostic data from 3 studies (16–18) involving 355 participants.A combined analysis demonstrated that patients with ILA was significantly correlated with shortened PFS (HR=1.59; 95% CI=1.08-2.32; P=0.017) than those without, with no significant heterogeneity identified between studies (I^2^ = 0%; P=0.772) (**Figure 3**).

### 3.4 ILA and disease-free survival

**Figure 4:**
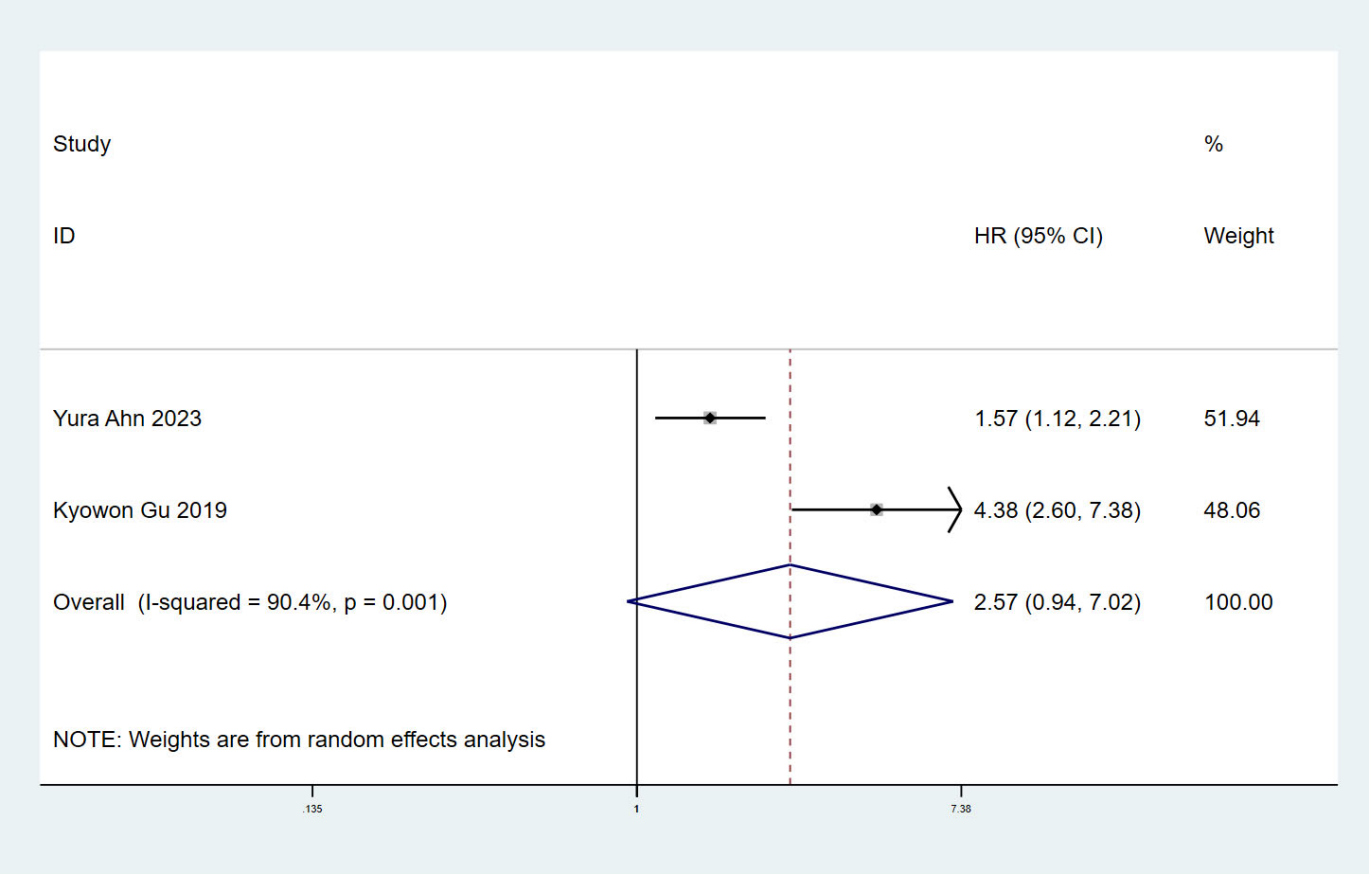
Forest plots of the association between ILA and the disease-free survival of patients with lung cancer.

There were 2 studies (7, 9) with a total of 1906 patients investigating the correlation between ILA and DFS. Significant heterogeneity was observed in the included studies (I^2^ = 90.4%, P = 0.001), so a random effects model was used. Interestingly, the results indicated that the combined ILA patients had no significant association with DFS (HR: 2.07, 95% CI: 0.94–7.02, P = 0.066) (**Figure 4**).

### 3.5 ILA and cancer-specific survival

**Figure 5:**
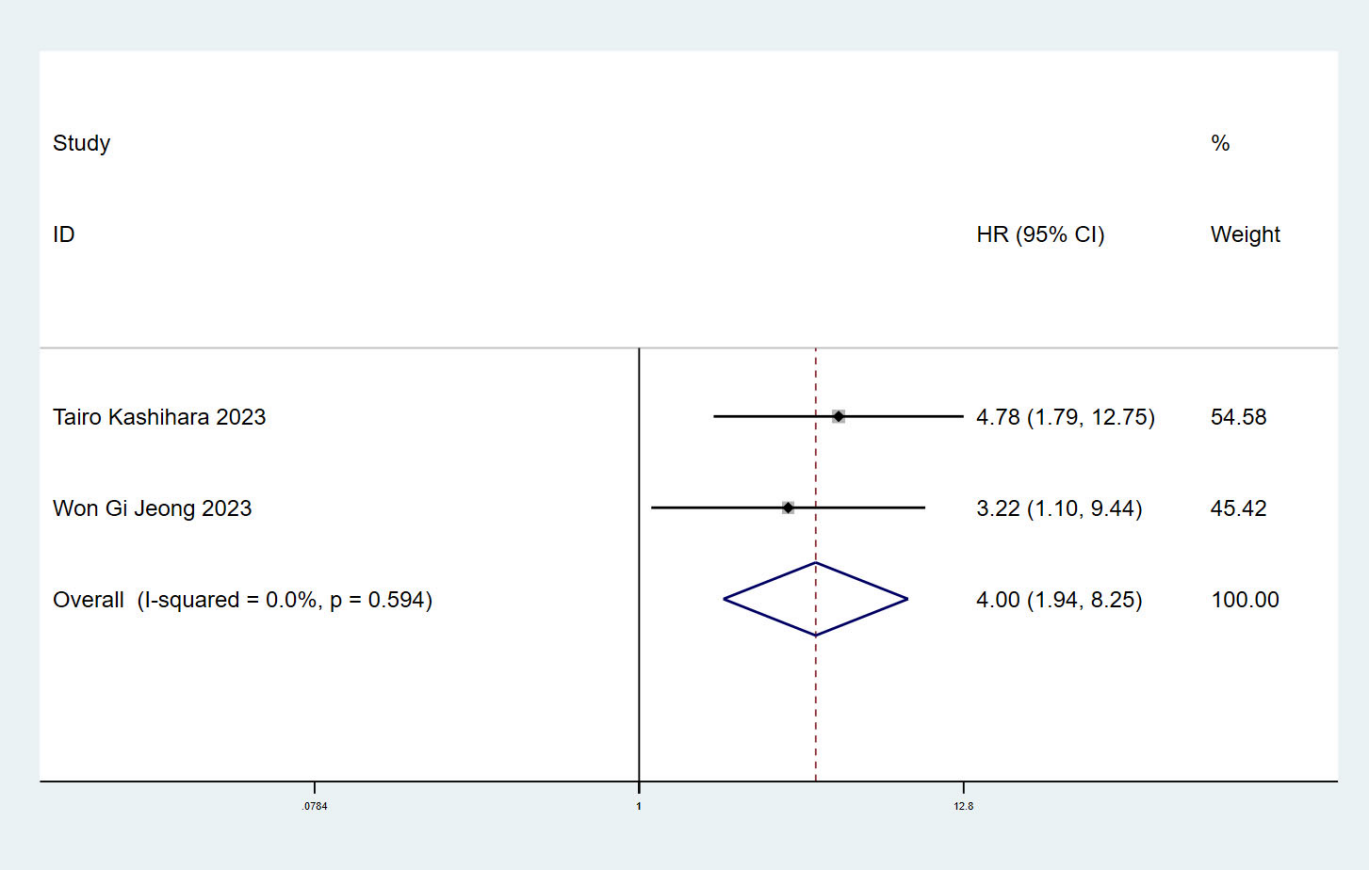
Forest plots of the association between ILA and the cause-specific mortality of patients with lung cancer.

The association between ILA and CSS in cancer patients was explored in two articles (12, 17) with 341 individuals. We found significant correlation between ILA and CSS in lung cancer patients (HR=4.00, 95% CI: 1.94-8.25, P<0.001) using a fixed effect model (I^2^ = 0%, P= 0.594) (**Figure 5**).

### 3.6 Association of ILA with clinicopathological characteristics

Of the articles included for analysis in the present study, a total of 8 studies, involving 2330 patients with lung cancer, reported a relationship between the ILA and clinicopathological factors of lung cancer. As shown by the combined results in **Table 3**, patients with ILA were significantly associated with male (OR= 2.43; 95% CI= 1.48-3.98; P<0.001), smoking history (OR= 2.11; 95% CI=1.37-3.25; P<0.001), advanced age (OR=2.50; 95% CI= 1.56-4.03; P<0.001), squamous carcinoma (OR=0.42; 95% CI=0.24-0.71; P=0.01), and EGFR mutation (OR=0.50; 95% CI=0.32-0.78; P=0.002). There was no significant correlation, however, between the ILA patients and Race (OR=1.92; 95% CI= 0.51-7.18; P= 0.334), Stage (OR= 0.93; 95% CI= 0.65-1.34; P= 0.708), ALK (OR= 0.30; 95% CI= 0.11-1.38; P= 0.144) (**Supplementary Figures S6–13**).

**Table 3:** The correlation between ILA and clinicopathological characteristics in patients with lung cancer.

Clinicopathological features	Numbers of Studies	Heterogeneity		OR(95%CI)	P value	Effect model
		I²(%)	Ph			
Gender (male vs female)	8	53.1	0.037	2.43 (1.48, 3.98)	<0.001	Random
Smoking status (used vs never)	7	15.9	0.309	2.11 (1.37, 3.25)	<0.001	Fixed
Age (old vs young)	2	17.8	0.27	2.50 (1.56, 4.03)	<0.001	Fixed
Race (white vs other)	2	0	0.403	1.92 (0.51, 7.18)	0.334	Fixed
Pathological type (adenocarcinoma vs non-adenocarcinoma)	7	61.1	0.017	0.42 (0.24, 0.71)	0.01	Random
Stage (I-IIIA vs IIIB-IV)	3	0	0.849	0.93 (0.65, 1.34)	0.708	Fixed
EGFR (Mutated vs Wild)	2	0	0.404	0.50 (0.32, 0.78)	0.002	Fixed
ALK (Rearranged vs Wild)	2	0	0.878	0.30 (0.11, 1.38)	0.144	Fixed

OR , Odds ratio; CI ,  confidence interval; Ph , P values of Q test for heterogeneity test.

### 3.7 Sensitivity analysis

**Figure 6:**
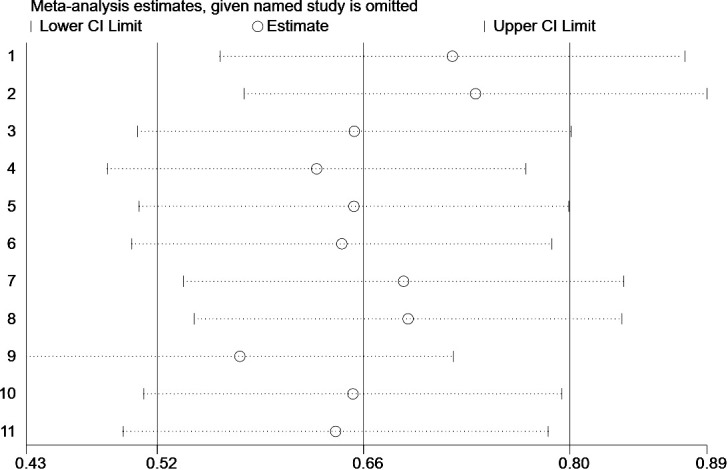
Sensitivity analysis of the results of overall survival analysis.

**Figure 7:**
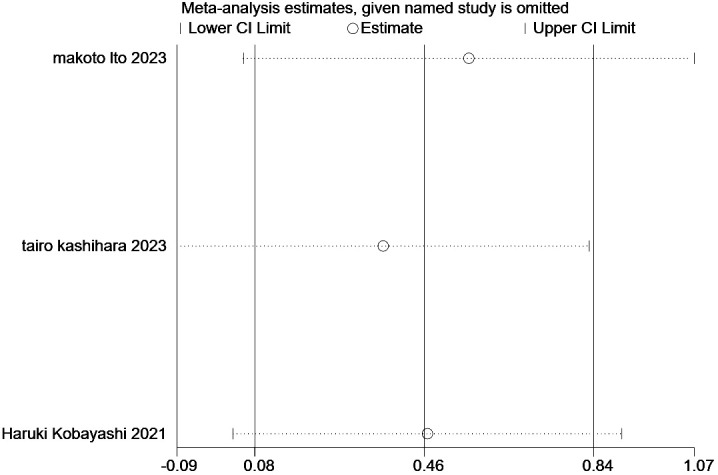
Sensitivity analysis of the results of progression-free survival analysis.

In the present study, we performed a sensitivity analysis on the relationship between the ILA and OS (**Figure 6**) and PFS (**Figure 7**), through which we determined that the significance of ILA in predicting OS and PFS in patients with lung cancer did not change after eliminating any single article.

### 3.8 Publication bias

**Figure 8:**
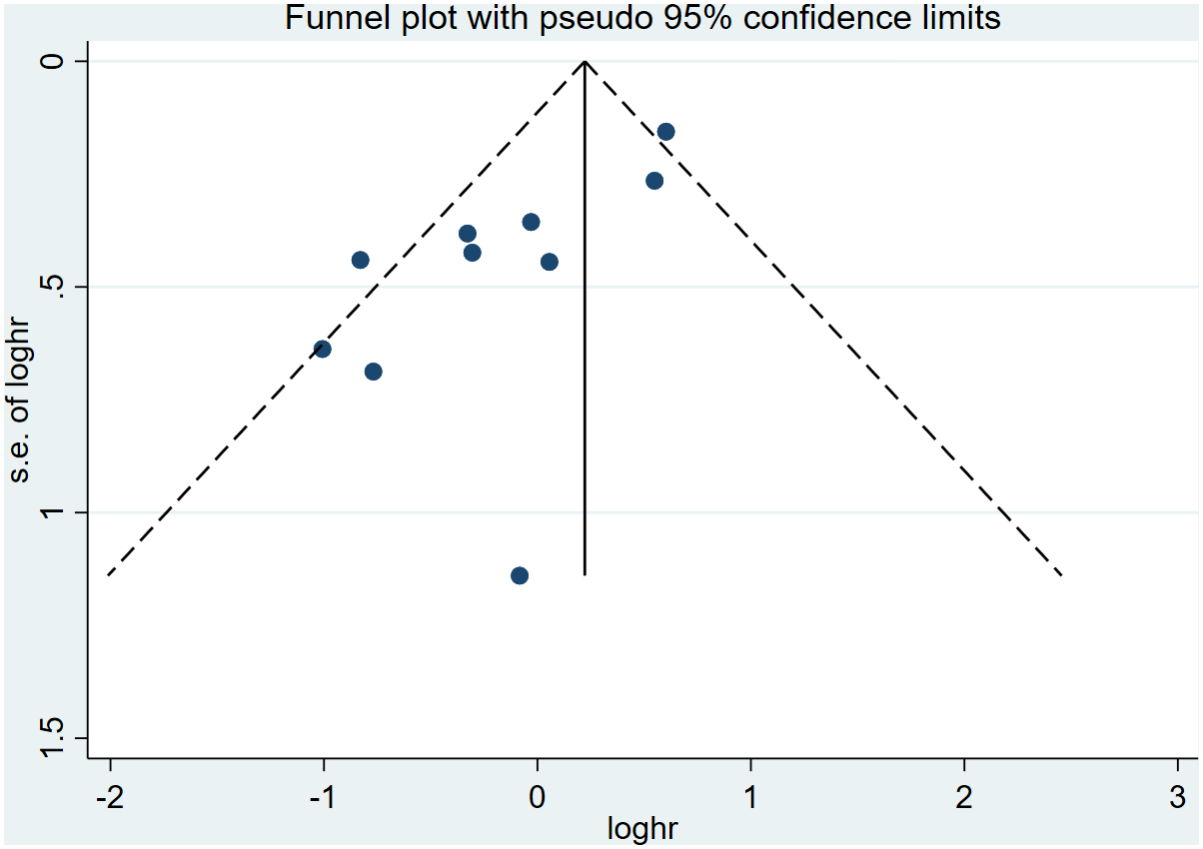
Funnel plots showing the publication bias of the included studies in the overall survival analysis.

**Figure 9:**
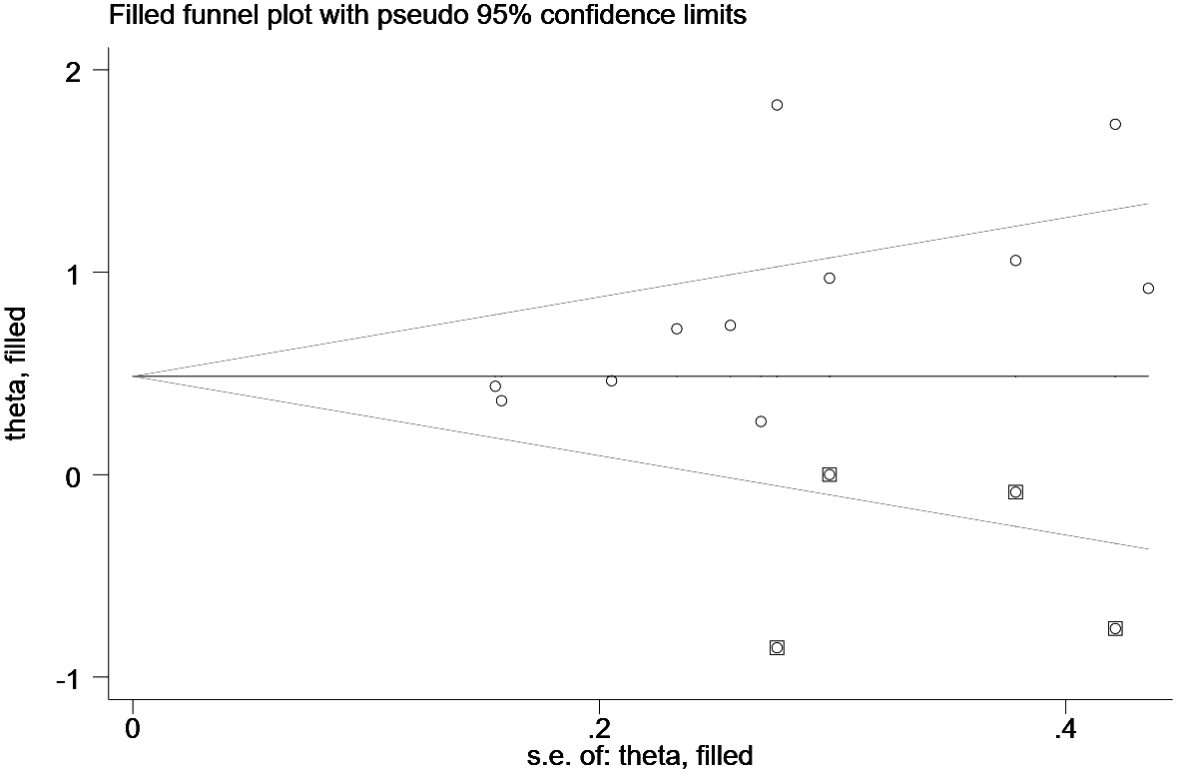
Funnel plot after Trim and Fill analysis.

Funnel plot and Egger’s test evaluated the inclusion for publication bias. For the Meta-analysis of the relationship between ILA and OS, the meta-analysis was biased (P = 0.04) (**Figure 8**); Next, the trim and fill method was utilized to calculate the number of missing studies in OS. By factoring in the missing hypothesis studies, the combined HR of OS was recalculated but was not substantially different. As a result, the publication bias had little impact, and the outcome was stable (**Figure 9**).

## 4 Discussion

Washko et al. (21, 22) first introduced the concept of interstitial pulmonary abnormalities (ILA) to assess interstitial changes in the lungs using high-resolution computed tomography (HRCT) in 2010. Subsequent research has shown that ILA is linked to the prognosis of lung cancer. Grantorser et al. (6) conducted meta-analysis revealed that ILA is associated with a higher risk of lung cancer and increased mortality among ILA patients. Additionally, Tepatel et al. (23) conducted an extensive cohort study demonstrated that ILA is linked to the progression of interstitial lung disease (ILD) and postoperative complications in lung cancer patients. These findings suggest that ILA could be a valuable marker for predicting poor outcomes in lung cancer and may indicate an increased risk of the disease, serving as a prognostic indicator.

In this meta-analysis, we aimed to assess the prognostic significance of ILA in lung cancer. The study included 12 research articles with a total of 4248 cases. Our findings showed that individuals with ILA face a heightened risk of OS, PFS, and CSS along with poorer combined HRs. Our meta-analysis did not uncover the predictive significance of ILA for DFS due to the constraints resulting from a limited sample size, leading to decreased statistical efficiency. This issue may stem from inadequate statistical power, given that only 2 studies examined DFS in relation to ILA. Additional investigations are essential to explore the predictive value of ILA for DFS in lung cancer patients and to aid in risk assessment for disease management and survival. Subsequent large-scale prospective studies are necessary to confirm our conclusions. The correlation between ILA and OS was consistent across various factors such as treatment approaches, geographical regions, tumor types, sample sizes, and analytical methods. However, Higo et al. (15) reported no significant difference in 2-year OS between lung cancer patients with ILA and those without ILA, possibly due to the previous ambiguity in defining ILA. On the other hand, Kobayashi et al. (18) Ito et al. (16) and Kashihara et al. (17) discovered that ILA was not an independent prognostic factor for PFS in lung cancer patients undergoing chemoradiotherapy. Interestingly, combined results indicated that patients with ILA had a poorer PFS (HR =1.55, 95% CI: 0.67-3.16; P=0.258). Several potential explanations for these conflicting outcomes have been suggested, such as the inclusion of only lung cancer patients receiving chemoradiotherapy in these studies, thereby excluding some ILA patients in good health, as well as the limited sample size of the studies.

Moreover, ILA was found to be significantly associated with such clinicopathologic features as male, advanced age, smoking history, squamous cell carcinoma, and EGFR mutated, which suggests that male, advanced age, smoking history, squamous cell carcinoma, and EGFR mutated patients with lung cancer tend to suffer from ILA. The content of these risk factors consists of clinical information readily available in daily clinical practice. It will help develop treatment and prevention strategies for this hidden disease and could provide very useful information for clinicians to improve outcomes for lung cancer patients.

However, the mechanism between ILA and the prognosis of lung cancer has not been elucidated. Previous studies suggest that ILA may represent an early stage of pulmonary fibrosis (PF) in unknown interstitial lung disease (ILD) (4). Research has shown that fibrotic lung disease and lung cancer may share common pathobiological processes, such as genetic alterations (24), epigenetic similarities (including DNA methylation and altered mRNA expression profiles) (24), as well as abnormalities in intercellular communication, alterations in intracellular signaling pathways (25), increased programmed cell death-ligand 1 (PD-L1) signaling (26) and overexpression of transforming growth factor (TGF)-β molecules (27, 28). These factors create a conducive microenvironment for cancer growth and invasion, thus indicating that patients with ILA are at a higher risk for a poor prognosis.

However, it is important to acknowledge the limitations of this meta-analysis. Firstly, all the studies reviewed were retrospective in nature, which raises concerns about potential bias. Secondly, the studies we included were mainly from the United States and Asia, and there was a lack of studies on Caucasian and African populations. Thirdly, the limited number of eligible studies prevented a thorough analysis of potential sources of heterogeneity. Lastly, publication bias could not be completely ruled out, as studies with positive results may have a higher likelihood of being published.

In summary, ILA lung cancer patients are associated with poorer OS, RFS and CSS, so ILA can be used as a potential predictor of lung cancer prognosis.

## Data availability statement

The original contributions presented in the study are included in the article/**Supplementary Material**. Further inquiries can be directed to the corresponding author.

## Author contributions

XT: Conceptualization, Data curation, Resources, Software, Visualization, Writing – original draft, Writing – review & editing, Methodology. YS: Conceptualization, Data curation, Investigation, Validation, Writing – original draft, Writing – review & editing. XG: Data curation, Resources, Software, Writing – original draft, Methodology, Visualization. SY: Conceptualization, Data curation, Investigation, Validation, Writing – original draft. WZ: Formal analysis, Resources, Project administration, Supervision, Writing – original draft, Writing – review & editing.
