# Supplementary material for: Prognostic impact of interstitial lung abnormalities in lung cancer: a systematic review and meta-analysis
Source: Front Oncol. 2024 May 10;14:1397246. doi: 10.3389/fonc.2024.1397246 (PMC11116699; doi:10.3389/fonc.2024.1397246)
Supplement: Supplementary file 1 [file DataSheet_1.pdf]

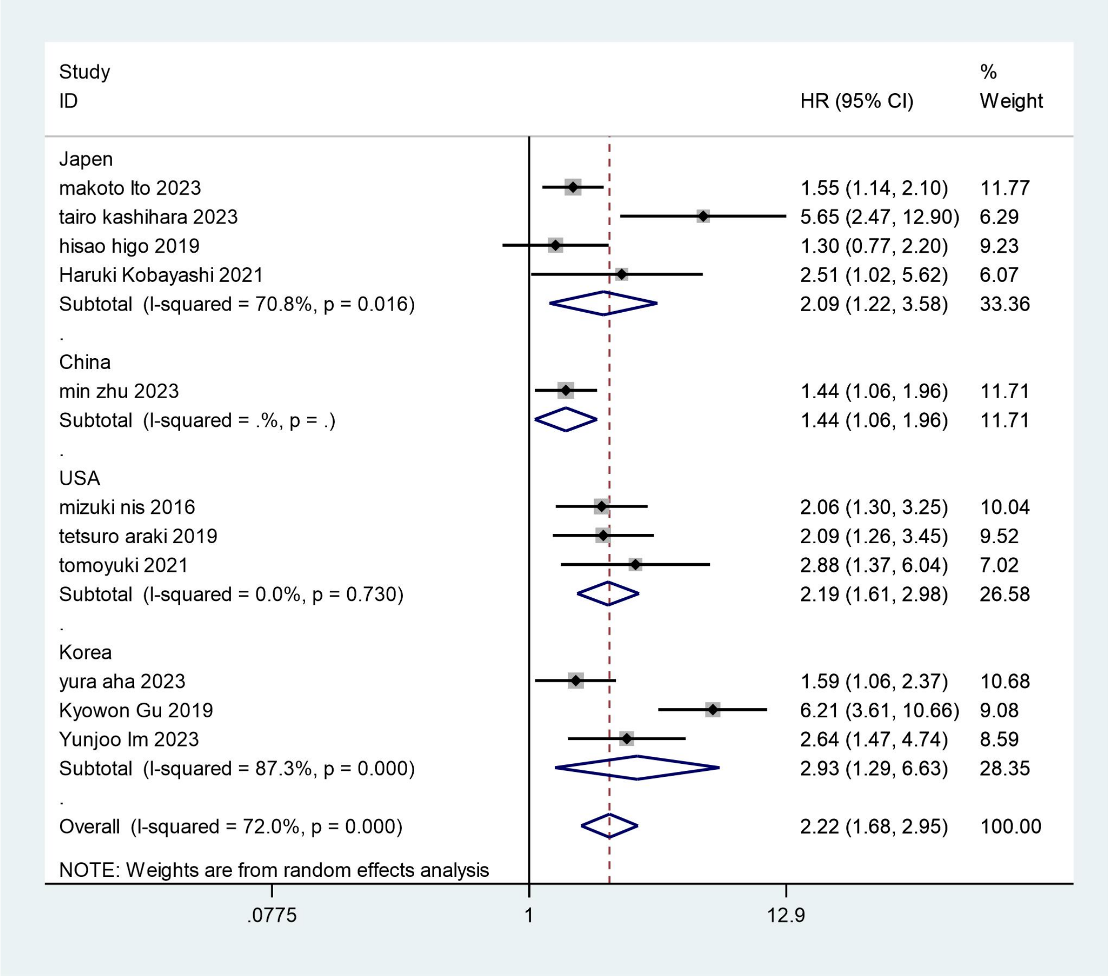

Figure S1. Forest plots of 11 studies examining the association between ILA and the OS of patients with lung cancer in the subgroup of study region.

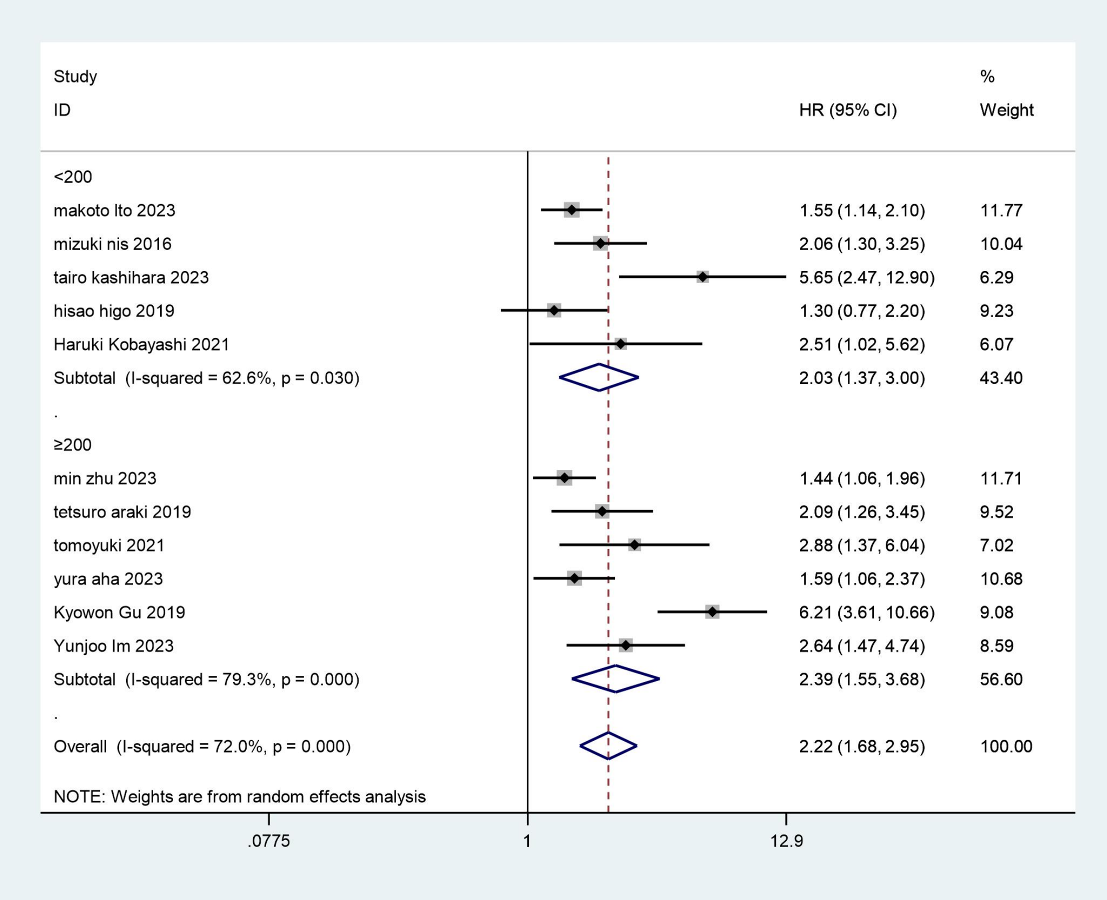

Figure S2. Forest plots of 11 studies examining the association between ILA and the OS of patients with lung cancer in the subgroup of sample size.

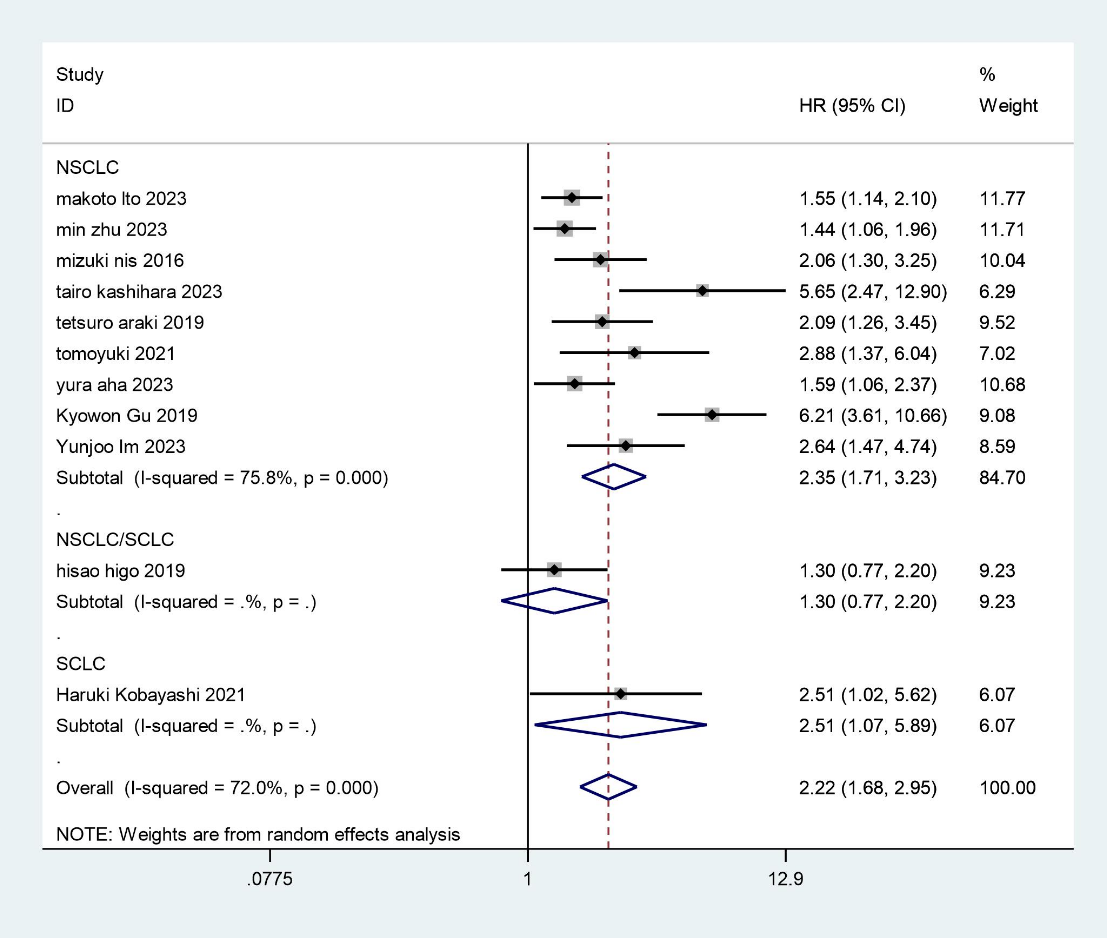

Figure S3. Forest plots of 11 studies examining the association between ILA and the OS of patients with lung cancer in the subgroup of cancer types.

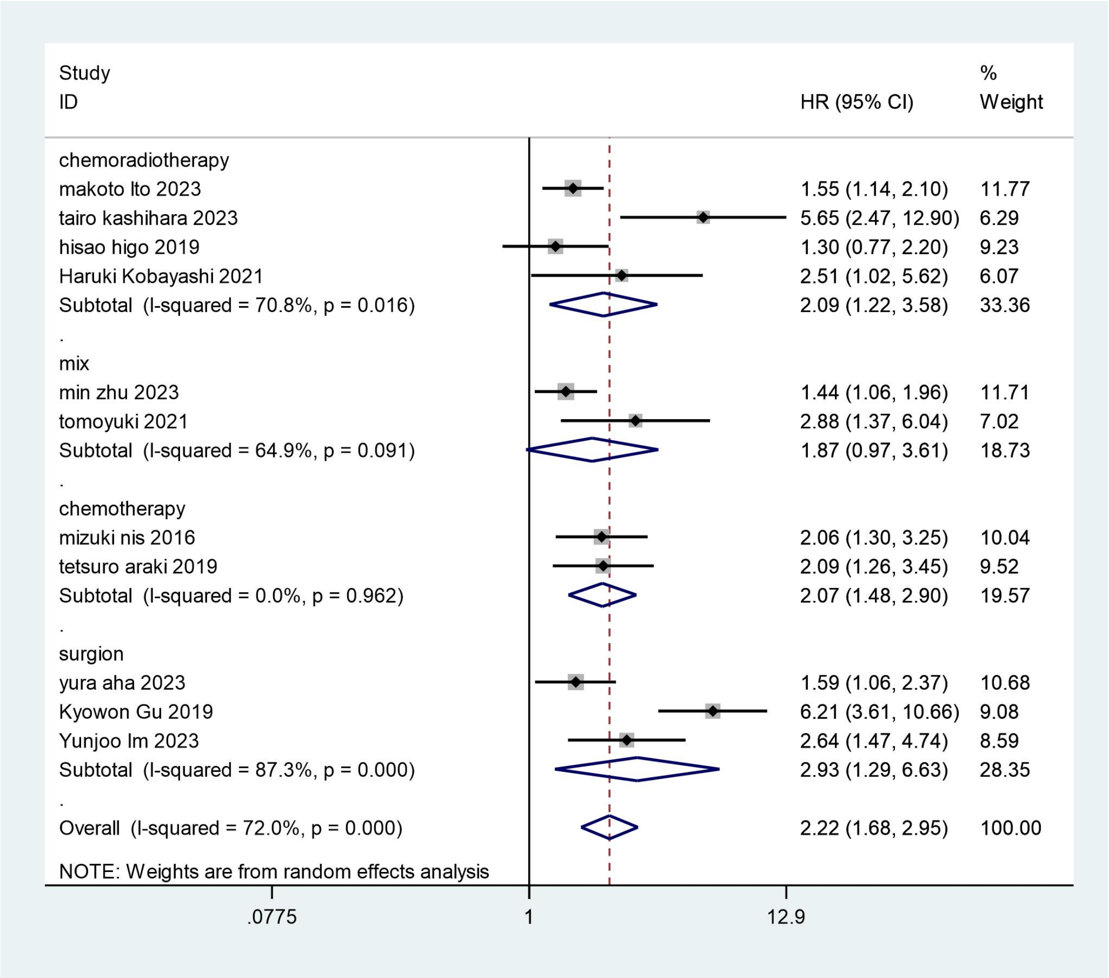

Figure S4. Forest plots of 11 studies examining the association between ILA and the OS of patients with lung cancer in the subgroup of treatment.

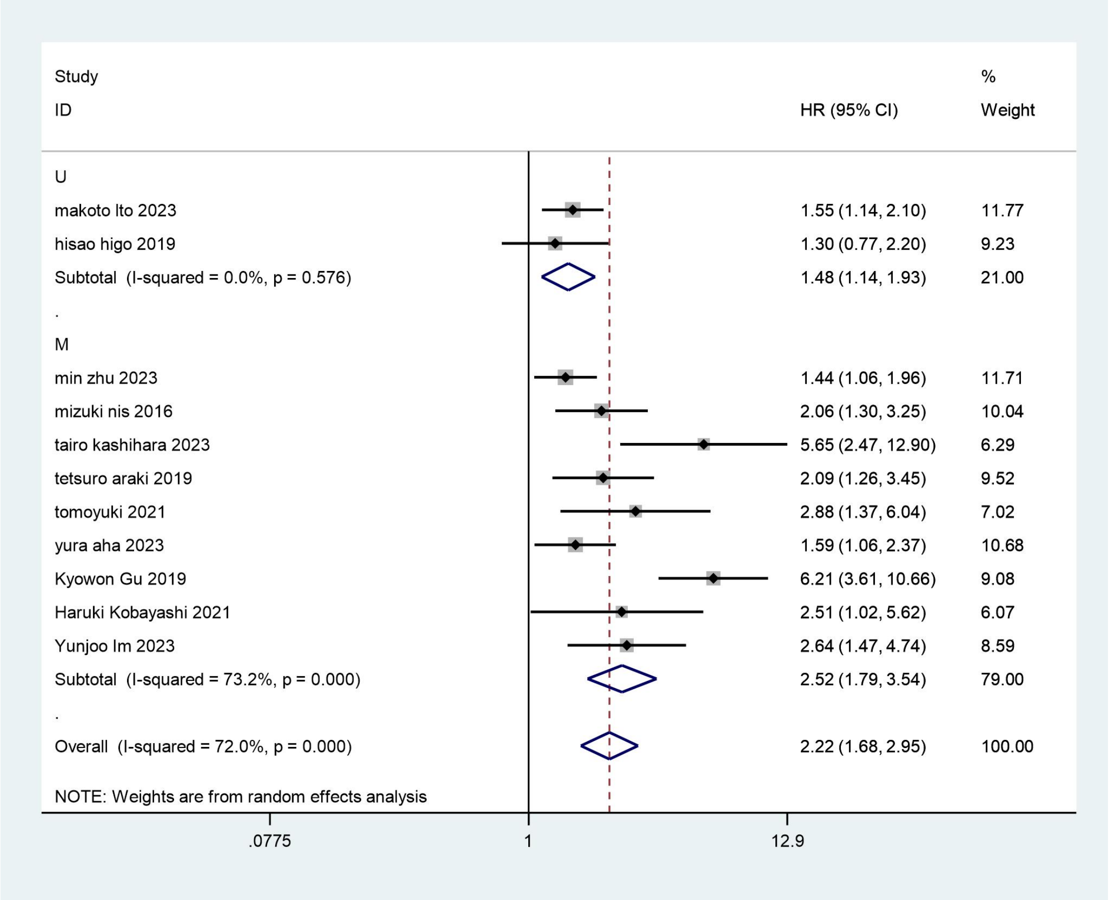

Figure S5. Forest plots of 11 studies examining the association between ILA and the OS of patients with lung cancer in the subgroup of analysis methods.

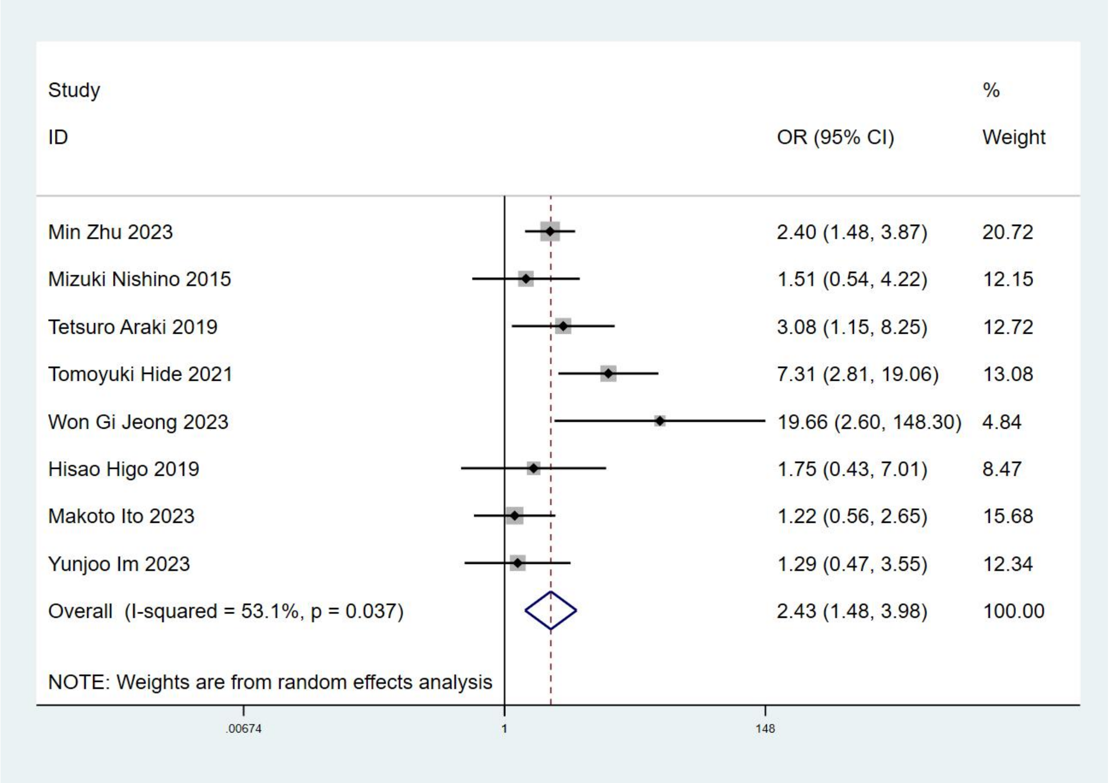

Figure S6. Forest plots of 8 studies examining the association between ILA and gender of patients

with lung cancer.

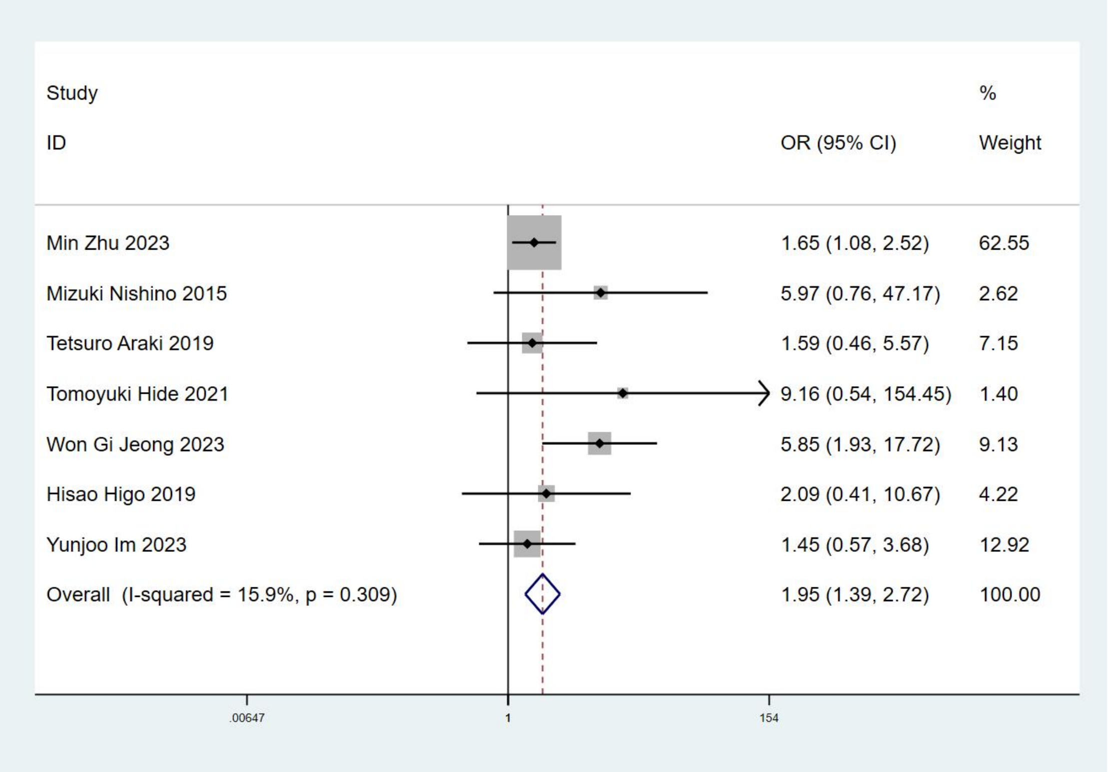

Figure S7. Forest plots of 7 studies examining the association between ILA and smoking status of patients with lung cancer.

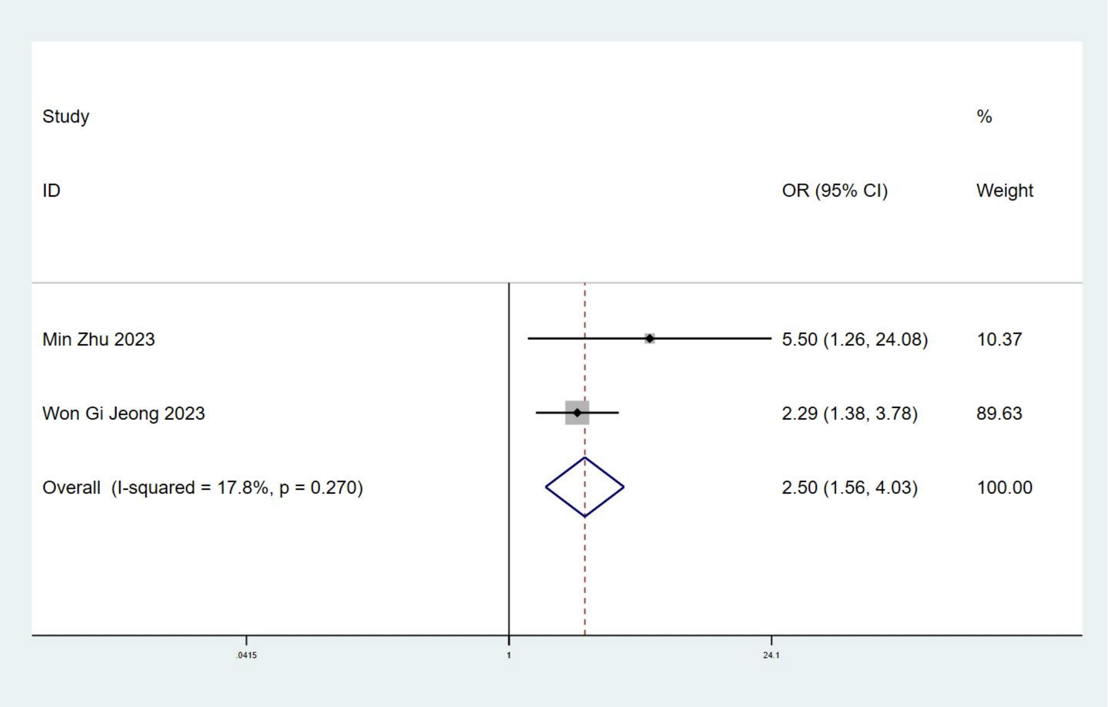

Figure S8. Forest plots of 2 studies examining the association between ILA and age of patients with lung cancer.

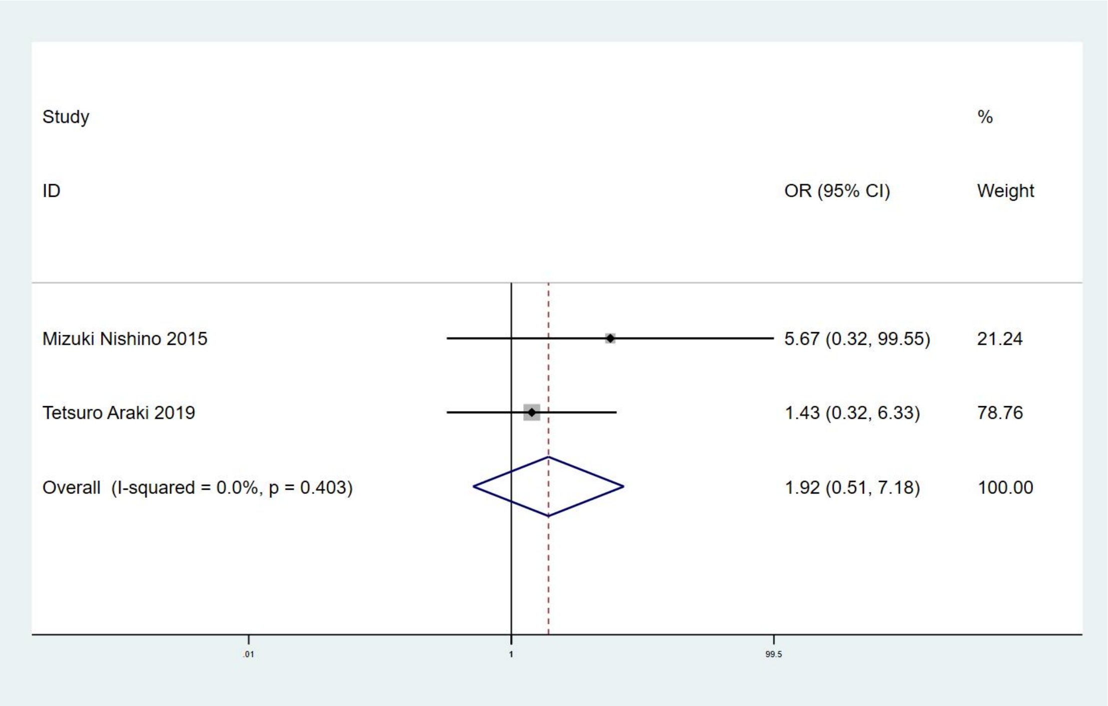

Figure S9. Forest plots of 2 studies examining the association between ILA and race of patients with lung cancer.

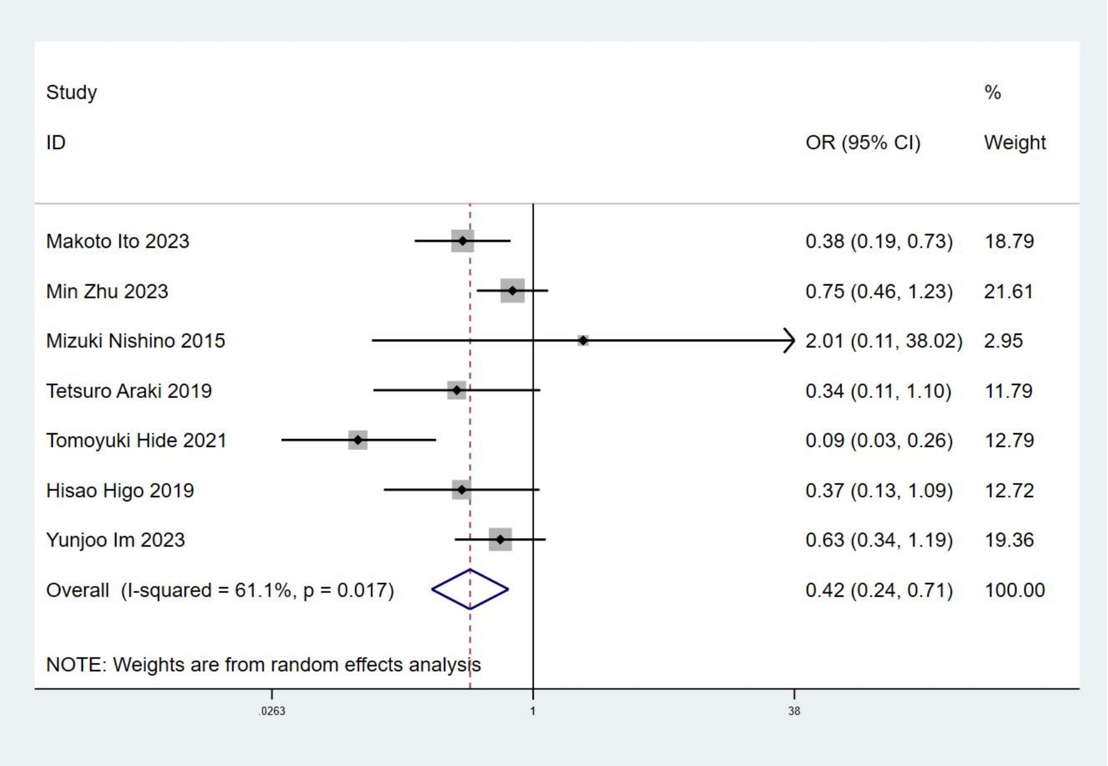

Figure S10. Forest plots of 7 studies examining the association between ILA and pathological type of patients with lung cancer.

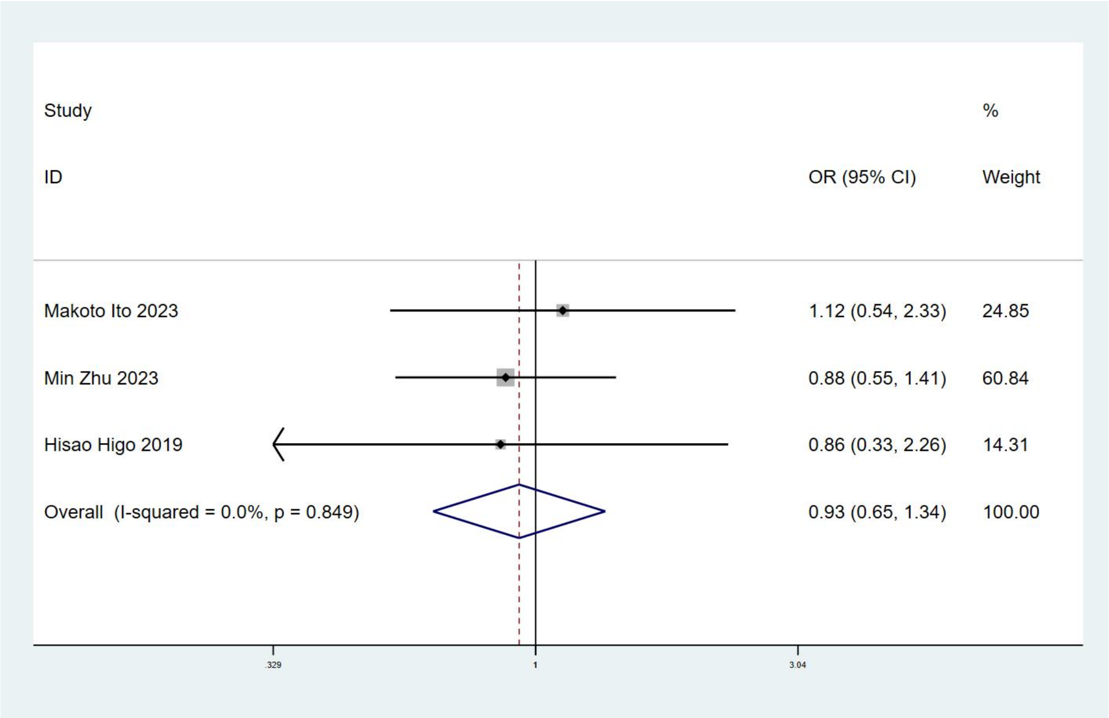

Figure S11. Forest plots of 3 studies examining the association between ILA and stage of patients with lung cancer.

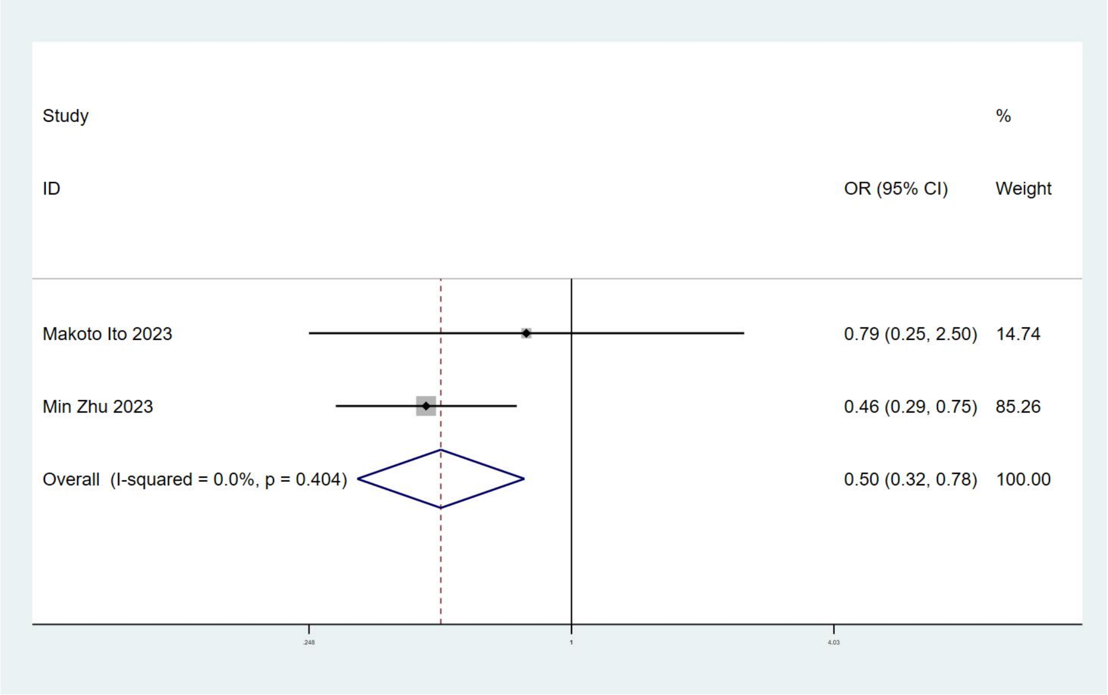

Figure S12. Forest plots of 2 studies examining the association between ILA and EGFR of patients with lung cancer.

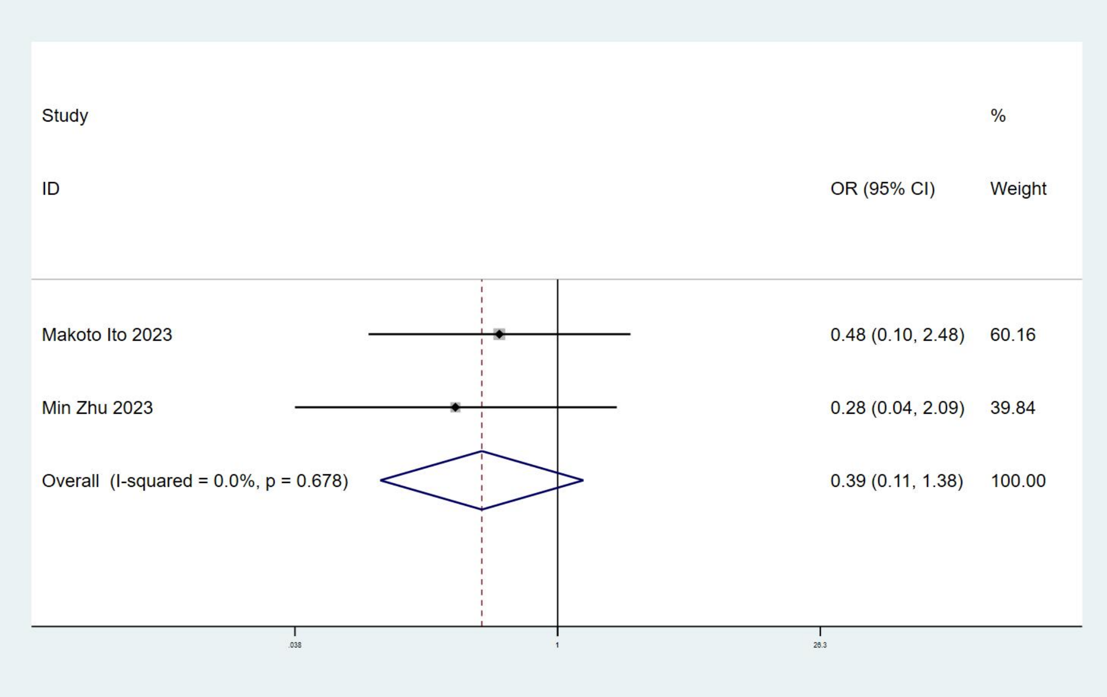

Figure S13. Forest plots of 2 studies examining the association between ILA and ALK of patients with lung cancer.
