# Supplementary material for: Prognostic impact of interstitial lung abnormalities in lung cancer: a systematic review and meta-analysis
Source: Front Oncol. 2024 May 10;14:1397246. doi: 10.3389/fonc.2024.1397246 (PMC11116699; doi:10.3389/fonc.2024.1397246)
Supplement: Supplementary file 2 [file Table_1.docx]

**Pubmed**

| **Search Number** | **Search Strategy** |
| --- | --- |
| #1 | Pulmonary Neoplasms[MeSH Terms] |
| #2 | (((((((((((((((Pulmonary Neoplasms[Title/Abstract]) OR (Neoplasms, Lung[Title/Abstract])) OR (Lung Neoplasm[Title/Abstract])) OR (Neoplasm, Lung[Title/Abstract])) OR (Neoplasms, Pulmonary[Title/Abstract])) OR (Neoplasm, Pulmonary[Title/Abstract])) OR (Pulmonary Neoplasm[Title/Abstract])) OR (Lung Cancer[Title/Abstract])) OR (Cancer, Lung[Title/Abstract])) OR (Cancers, Lung[Title/Abstract])) OR (Lung Cancers[Title/Abstract])) OR (Pulmonary Cancer[Title/Abstract])) OR (Cancer, Pulmonary[Title/Abstract])) OR (Cancers, Pulmonary[Title/Abstract])) OR (Pulmonary Cancers[Title/Abstract])) OR (Cancer of the Lung[Title/Abstract])) OR (Cancer of Lung[Title/Abstract]) |
| #3 | ((((ILA[Title/Abstract]) OR (ILAs[Title/Abstract])) OR (Interstitial lung abnormality[Title/Abstract])) OR (Interstitial lung abnormalities[Title/Abstract])OR (subclinical ILD[Title/Abstract]))OR (preclinical ILD[Title/Abstract]))OR (early ILD[Title/Abstract])) OR (abnormal CT finding)) OR (early interstitial lung)) OR (early phase interstitial change)) OR (early parenchymal lung disease) |
| #4 | #1 OR #2 |
| #5 | Prognosis[MeSH Terms] |
| #6 | (Prognoses[Title/Abstract]) OR (Prognostic Factors[Title/Abstract]) |
| #7 | #6 OR #7 |
| #8 | ((Computed Tomography[Title/Abstract]) OR (multidetector computer tomography[Title/Abstract])) OR (High resolution computer tomography[Title/Abstract]) |
| #9 | #3 AND #4 |
| #10 | #7 AND #8 |
| #11 | #9 AND #10 |

**Embase**

| **Search Number** | **Search Strategy** |
| --- | --- |
| #1 | 'lung neoplasm'/exp |
| #2 | 'pulmonary neoplasms':ab,ti OR 'neoplasms, lung':ab,ti OR 'lung neoplasm':ab,ti OR 'lung neoplasm':ab,ti OR 'neoplasm, lung':ab,ti OR 'neoplasms, pulmonary':ab,ti OR 'neoplasm, pulmonary':ab,ti OR 'pulmonary neoplasm'/exp OR 'pulmonary neoplasm':ab,ti OR 'lung cancer'/exp OR 'lung cancer':ab,ti OR 'cancer, lung'/exp OR 'cancer, lung':ab,ti OR 'cancers, lung':ab,ti OR 'lung cancers':ab,ti OR 'pulmonary cancer'/exp OR 'pulmonary cancer':ab,ti OR 'cancer, pulmonary':ab,ti OR 'cancers, pulmonary':ab,ti OR 'pulmonary cancers':ab,ti OR 'cancer of the lung'/exp OR 'cancer of the lung':ab,ti OR 'cancer of lung':ab,ti |
| #3 | #1 OR #2 |
| #4 | 'interstitial lung disease'/exp |
| #5 | 'ila':ab,ti OR 'ilas':ab,ti OR 'interstitial lung abnormality':ab,ti OR 'interstitial lung abnormalities':ab,ti OR 'subclinical ILD':ab,ti OR 'preclinical ILD':ab,ti OR 'early ILD':ab,ti OR 'subclinical ILD':ab,ti OR 'abnormal CT finding':ab,ti OR 'early interstitial lung':ab,ti OR 'early phase interstitial change':ab,ti OR 'early parenchymal lung disease':ab,ti |
| #6 | #4 OR #5 |
| #7 | 'Prognosis'/exp |
| #8 | 'Prognoses':ab,ti OR 'Prognostic Factors':ab,ti |
| #9 | #7 OR #8 |
| #10 | 'Computed Tomography':ab,ti OR 'multidetector computer tomography':ab,ti OR 'High resolution computer tomography':ab,ti |
| #11 | #3 AND #6 |
| #12 | #9 AND #10 |
| #13 | #11 AND #12 |

**Cochrane**

| **Search Number** | **Search Strategy** |
| --- | --- |
| #1 | MeSH descriptor: [Pulmonary Neoplasms] explode all trees |
| #2 | (Pulmonary Neoplasms):ti,ab,kw OR (Neoplasms, Lung):ti,ab,kw OR (Lung Neoplasm):ti,ab,kw OR (Neoplasm, Lung):ti,ab,kw OR (Neoplasms, Pulmonary):ti,ab,kw OR (Neoplasm, Pulmonary):ti,ab,kw OR (Pulmonary Neoplasm):ti,ab,kw OR (Lung Cancer):ti,ab,kw OR (Cancer, Lung):ti,ab,kw OR (Cancers, Lung):ti,ab,kw OR (Lung Cancers):ti,ab,kw OR (Pulmonary Cancer):ti,ab,kw OR (Cancer, Pulmonary):ti,ab,kw OR (Cancers, Pulmonary):ti,ab,kw OR (Pulmonary Cancers):ti,ab,kw OR (Cancer of the Lung):ti,ab,kw OR (Cancer of Lung |
| #3 | #1 OR #2 |
| #4 | MeSH descriptor: [interstitial lung abnormality] explode all trees |
| #5 | (ILA):ti,ab,kw OR (Interstitial lung abnormalities):ti,ab,kw OR (ILAs):ti,ab,kw OR (ILAS):ti,ab,kw AND (Interstitial lung abnormalitie):ti,ab,kw OR (subclinical ILD):ti,ab,kw OR (preclinical ILD):ti,ab,kw OR (early ILD):ti,ab,kw OR (abnormal CT finding):ti,ab,kw OR (early interstitial lung):ti,ab,kw OR (early phase interstitial change):ti,ab,kw OR (early parenchymal lung disease):ti,ab,kw |
| #6 | #4 OR #5 |
| #7 | MeSH descriptor: [Prognosis] explode all trees |
| #8 | (Prognoses):ti,ab,kw OR (Prognostic Factors):ti,ab,kw |
| #9 | #6 OR #7 |
| #10 | (Computed Tomography):ti,ab,kw OR (multidetector computer tomography):ti,ab,kw OR (High resolution computer tomography):ti,ab,kw |
| #11 | #3 AND #6 |
| #12 | #9 AND #10 |
| #13 | #11 AND #12 |

**Web Of Science**

| **Search Number** | **Search Strategy** |
| --- | --- |
| #1 | ((((((((((((((((TS=(Pulmonary Neoplasms)) OR TS=(Neoplasms, Lung)) OR TS=(Lung Neoplasm)) OR TS=(Neoplasm, Lung)) OR TS=(Neoplasms, Pulmonary)) OR TS=(Neoplasm, Pulmonary)) OR TS=(Pulmonary Neoplasm)) OR TS=(Lung Cancer)) OR TS=(Cancer, Lung)) OR TS=(Cancers, Lung)) OR TS=(Lung Cancers)) OR TS=(Pulmonary Cancer)) OR TS=(Cancer, Pulmonary)) OR TS=(Cancers, Pulmonary)) OR TS=(Pulmonary Cancers)) OR TS=(Cancer of the Lung)) OR TS=(Cancer of Lung) |
| #2 | (((((((((TS=(ILA) OR TS=(ILAs)) OR TS=(Interstitial lung abnormality)) OR TS=(Interstitial lung abnormalities))OR TS=(subclinical ILD))OR TS=(preclinical ILD))OR TS=(early ILD)) OR TS=(abnormal CT finding)) OR TS=(early interstitial lung)) OR TS=(early phase interstitial change)) OR TS=(early parenchymal lung disease) |
| #3 | ((TS=(Prognosis)) OR TS=(Prognoses)) OR TS=(Prognostic Factors) |
| #4 | ((TS=(Computed Tomography)) OR TS=(multidetector computer tomography)) OR TS=(High resolution computer tomography) |
| #5 | #1 AND #2 |
| #6 | #3 AND #4 |
| #7 | #5 AND #6 |
